# Resilience to trauma in the two largest cities of Brazil: a cross-sectional study

**DOI:** 10.1186/s12888-014-0257-0

**Published:** 2014-09-16

**Authors:** Liliane Vilete, Ivan Figueira, Sérgio Baxter Andreoli, Wagner Ribeiro, Maria Ines Quintana, Jair de Jesus Mari, Evandro Silva Freire Coutinho

**Affiliations:** Universidade Federal do Rio de Janeiro, Instituto de Psiquiatria, Av: Venceslau=Brás 71 – fundos – Botafogo CEP, Rio de Janeiro, 22290-140 Brazil; Universidade Católica de Santos, São Paulo, Brazil; Universidade Federal de São Paulo, Departamento de Psiquiatria, São Paulo, Brazil; King’s College London, Health Service and Population Research Department, Institute of Psychiatry, London, UK; Fundação Oswaldo Cruz, Escola Nacional de Saúde Pública, Rio de Janeiro, Brazil

**Keywords:** Psychological resilience, Positive affect, Coping behavior, Traumatic stress disorders

## Abstract

**Background:**

Resilience is a dynamic process involving the interaction between intrapsychic and social factors of risk and protection. For resilience to be recognized there must be a significant threat to the individual, such as a traumatic event, and a good quality of adjustment. The aim of this study was to identify predisposing factors and possible mechanisms associated with resilience to traumatic events in the general population.

**Methods:**

We conducted a cross-sectional study with a random sample, aged 15–75 years, living in the two largest cities in Brazil, who were exposed to trauma (N = 3,231). Positive adaptation to trauma was defined as the lifetime absence of anxiety (including posttraumatic stress disorder), depression and alcohol related disorders in the presence of at least one traumatic event. Logistic regression models predicting resilience were used to estimate the incidence density ratio. This measure expresses the extent to which the rate of resilience differs from the exposed group to the non-exposed group. Moreover, we explored the relationship between positive/negative affect and resilience, using linear regression models.

**Results:**

Male gender was a predisposing factor to positive adaptation (incidence density ratio [IDR] = 1.34; *p* < 0.001). There was an inverse linear relationship between childhood violence and resilience (IDR = 0.67; 0.53; 0.19; *p* < 0.001). Our findings suggest that the absence of parental mental disease (IDR = 1.35; *p* = 0.07) also predisposes individuals to positive adaptation.

**Conclusions:**

This study provides results that help to identify vulnerable groups and protective factors that may lead to a positive adaptation following traumatic experiences.

## Background

Potentially traumatic experiences that endanger the life or physical integrity of individuals and their loved ones are common, although they vary over time and among populations. In some localities, as in Zurich (Switzerland), the prevalence of these traumatic events is low, with only 28% of the population claiming to have experienced at least one traumatic event in their life [1]. By comparison, in Detroit (USA) the prevalence is as high as 89% of the adult population [2]. In the cities of Rio de Janeiro and São Paulo (Brazil) nearly 90% of individuals from 15 to 75 years old have faced lifetime traumatic events [3].

Epidemiological data indicate that about nine percent of individuals will develop posttraumatic stress disorder (PTSD) after experiencing a trauma [2-4]. PTSD is a syndrome that includes nightmares and intrusive thoughts about the traumatic event, avoidance of situations that recall the event, emotional numbing, and physiological hyperarousal that persist for at least a month. Besides PTSD, other disorders are commonly developed in the aftermath of a trauma, such as major depressive disorder and generalized anxiety disorder [5].

Fortunately, not all trauma-exposed people will develop mental disorders. The term resilience, with roots in the sciences of physics and mathematics, describes the ability of a material, when under a load, to store strain energy, and to bend elastically and bounce back without breaking [6]. This term was introduced in the field of Psychiatry in the late 1970s by scholars of developmental psychopathology. They observed children exposed to severe adversity in an effort to study early influences and pathways that led away from psychopathology [7-9]. The initial idea of “invulnerability” was replaced by the concept of resilience to correct the erroneous impression that the resistance to stress could be constitutional and absolute. According to Rutter [10], this resistance is relative and results from constitutional and environmental factors. Also, resilience should not be understood as a fixed or static quality, since it varies over time and according to circumstances.

The definition of resilience has little consensus in the literature, with substantial variations in its operationalization [11]. Masten states that two criteria are required to identify a process of resilience. First, there has to be a significant threat to the individual, such as a high-risk threat or exposure to severe adversity or a traumatic event. Second, the quality of adaptation must be good [8,12]. From the trauma perspective, resilience is an effective adaptation in the aftermath of significant threats to personal and physical integrity [13]. There is also little consensus about the measurement of key constructs, for example, concerning the definition of what positive adjustment means [11]. In the context of traumatic events, Hoge et al. [14] define resilient individuals as those persons who experience trauma and do not develop PTSD. Bonanno et al. [15] use the operational definition of resilience as the absence of PTSD symptoms or the presence of only one symptom of the disorder. However, other researchers have criticized this operationalization, asserting that resilience is more than the absence of PTSD, just as mental health is more than the absence of a mental disorder. They argue that it is inappropriate to consider the absence of PTSD as evidence of resilience, just as it would be inappropriate to consider the absence of fever (accurately measured using a thermometer) as evidence of good health in individuals, since one may have other symptoms of disease that cannot be measured by a thermometer [16].

Although numerous scales have been developed to measure resilience, it is not clear whether a resiliency scale (based on individual or social attributes) can truly measure improvement in resilience (e.g., good adaptation after a threat) [14]. Also, evidence of environmentally mediated risk and a quantitative measure of the degree of such risk is necessary to effectively study this construct, because apparent resilience may simply be a function of variation in risk exposures [17].

In the study of resilience, it is also important to include an examination of the different effects of stress [18]. Although stressful experiences may render individuals more susceptible to subsequent stressors, there is evidence that stress may enhance an individual’s resistance to new hardships. Thus, depending on the time of its occurrence, its duration, and its intensity, exposure can be positive or “inoculating” [17]. In a study of firefighters investigating job-related traumas, Regehr et al. [19] found that a higher number of traumatic exposures increased subjects’ sense of internal locus of control and self-efficacy. Thus, rather than a vulnerability factor for adverse posttraumatic sequelae, trauma exposure may represent a protective factor, if it is associated with an increased sense of mastery or growth. However, unlike other stressors that may have positive effects, and whose ultimate effects may depend on the additional influence of other risks, child abuse is described as “unambiguously negative”, and clearly, the continuous exposure to it is one the most deleterious environmental risk that exists [20].

An interaction between intrapsychic and social processes of risk and protection is involved in the dynamic process of resilience [11,17,21]. The protective processes refer to the influences that alter or improve a person’s response to stress. Their action may not be detectable in the absence of a stressor and may only play a role as a moderating effect activated by risk or in response to adversity; analogous to an automobile airbag or an immune system response [10-12]. Many factors have been described as protective factors of resilience, such as social support, humor, self-esteem, coping styles, and positive emotions (as gratitude, interest, love) [22,23].

There are distinct and complementary adaptive functions and physiological effects associated with positive and negative emotions. Negative emotions focus and narrow thoughts and actions to prepare the body for fight or flight [24]. Positive emotions however, may broaden one’s thoughts and actions, and build important personal resources, including coping resources [24]. Thus, positive emotions may enhance resilience to future adversities [24]. Fredrickson et al. [25] suggest that positive emotions in the aftermath of crises buffer resilient people against depression and enhance recovery, by finding positive meaning in negative circumstances. Moreover, they undo the cardiovascular reactivity of negative emotions triggered by stress, promoting the recovery from these hyper-reactive states [26,27].

The study of resilience contributes to the understanding of the variability of responses to adversity exhibited by individuals, and allows expansion of prevention strategies, health promotion and treatment of mental disorders [10,17]. As far as we know, there is no epidemiological study in Brazil addressing resilience to traumatic events among adults in the general population.

The aim of this study was to investigate factors associated with positive adaptation to traumatic events in the general population. Our hypotheses are that resilience is influenced by individual and social characteristics - as gender, ethnic group, parental mental disorders, childhood trauma -, and that both positive and negative affect are related to positive adaptation to trauma.

## Methods

### Participants and design

The participants in this study came from a survey conducted with a representative sample of subjects, aged 15 to 75 years old, living in the two largest cities in Brazil: São Paulo and Rio de Janeiro. In 2006, the city of São Paulo had approximately 11 million inhabitants while Rio de Janeiro had 6 million. A multistage cluster sampling scheme was performed to obtain the sample. In the first stage, seven strata within the two cities were created and ranked according to their homicide rates. Then, all the census sectors within each stratus were mapped and randomly selected (second stage). In the third stage, within each census sector, we randomly selected 43 households (Sao Paulo) or 30 households (Rio de Janeiro). All residents aged 15 to 75 years from each included household were enumerated, and one of them was chosen based on Kish’s method [28]. Given an expected refusal rate of 20%, the estimated sample size was determined to be 1,500 interviews in Rio de Janeiro and 3,000 in Sao Paulo. We oversampled the most violent strata in Sao Paulo to identify more current PTSD cases to be referred to a case–control study and a clinical trial [3,29].

The present study was restricted to all individuals who experienced traumatic events (N = 3231), as listed in the Composite International Diagnostic Interview (CIDI 2.1). We added another 21 events as described by Ribeiro et al. [3]. Participants were asked to choose the worst event among those they had experienced. The symptoms of PTSD were investigated in relation to their worst trauma.

### Procedures

Data collection was carried out by a company specialized in household surveys, the Brazilian Institute of Public Opinion and Statistics (IBOPE). IBOPE provided the interviewers, the physical structure and logistic support for the training, management and supervision of the fieldwork. The supervisors re-interviewed at least 20% of all the participants to double-check the accuracy of interviewers’ work for quality control. Two of the authors (MIQ and WR) were responsible for training and following the fieldwork team.

### Measures and covariates

The interview included a number of fully structured questionnaires and scales related to psychiatric diagnoses, demographic variables, psychological traits and violence history [29]. A set of variables was derived from the original study to be included in the present analysis:

1. Resilience. According to what was discussed in the background section, and in the absence of any universal operationalization of this construct [11], we decided to define a resilient person as one who had a history of experiencing traumatic events, yet never presented one of the following psychiatric diagnoses (current or lifetime; by DSM-IV or ICD-10): (i) PTSD; (ii) phobic and other anxiety disorders (specific phobias, social phobia, agoraphobia, panic disorder, obsessive-compulsive disorder and generalized anxiety disorder); (iii) depressive disorders (episodic, recurrent and dysthymia); (iv) alcohol abuse and dependence. For diagnoses, we used the Brazilian version of the Composite International Diagnostic Interview (CIDI 2.1) validated by Quintana et al. [30]. We expanded the definition usually restricted to the lack of PTSD symptoms to the absence of a wider spectrum of mental problems that may be developed in the aftermath of a trauma. Thus, avoid classifying someone erroneously as resilient who may be in fact ill with a diagnosis other than PTSD was avoided.
  Erroneously classifying as non-resilient someone with symptoms that do not warrant a diagnosis and do not need treatment was also avoided. This was done using the DSM-IV criteria that considers that the disturbance may cause clinically significant distress or impairment in social, occupational, or other important areas of functioning and may have a minimal duration to warrant a diagnosis. There is a recent trend to define resilience as not only the absence of negative outcomes, but also as presence of positive ones (e.g., well-being). Participants were classified as resilient and non-resilient based on the absence and presence of psychopathology. To investigate the convergent validity of our classification with a positive outcome, scores of items on well-being between the resilient and non-resilient groups were compared.

2. Positive and Negative Affect. Affect was measured with the Positive and Negative Affect Schedule – PANAS [31]. PA and NA items are conceived as psychological-dispositional dimensions reflecting an individual’s proneness to experience life events as joyful or as distressing, respectively [32-34]. The instrument comprises 10 items of each dimension (PA and NA), with each item rated on a 5-point Likert scale (“not at all or very slightly” to “very much”). Interviewers asked participants the extent to which they experienced each particular emotion in their life as a whole, yielding trait-oriented scores. We summed the items separately for positive and negative affect subscales. Although originally designed as a self-report questionnaire, we administrated a Brazilian version of PANAS via face-to-face interviews to allow standardization of data gathering procedures regardless of the literacy level of the participant. An analysis of our survey sample showed that the structure and reliability of the Brazilian version of the PANAS are consistent with those of its original scale [35].

3. Subjective Well-being. Six items from the Subjective Well-Being Inventory (SUBI) [36] were used to measure subjective well-being; each item was rated on a 3-point Likert scale: “Do you feel your life is interesting (very much; to some extent; not so much)”; “Do you normally accomplish what you want to? (most of the time; sometimes; hardly ever)”; “How do you feel about the extent to which you have achieved success and are getting ahead? (very good; quite good; not so good)”; “Compared with the past, do you feel your present life is (very happy; quite happy; not so happy)?”; “Do you sometimes experience moments of intense happiness? (quite often; sometimes; hardly ever)”; “Do you sometimes experience a feeling of being part of mankind as of one large family? (quite often; sometimes; hardly ever)”. As the SUBI was not applied in its original format (we used just a few items of the scale), we did not compute a total score but, instead, we analysed each question separately.
4. Independent variables. Because the main objective of this study was to investigate etiological hypotheses from cross-sectional data, and because causal inference cannot be made *inter alia* in the presence of reverse causal relationships, our independent variables were restricted to events likely to have occurred before the onset of the mental disorder of those receiving a psychiatric diagnosis. The effects of the following variables were investigated: gender, ethnic group, mental disorder among parents (in at least one), childhood traumatic event (occurring before 13 years of age; with three categories: 1) ‘childhood trauma’ - with no physical or sexual abuse -; 2) ‘physical abuse’ - if at least one physical abuse and no sexual abuse; and 3) ‘sexual abuse’ - if at least one sexual abuse).
5. Covariates. In order to control for potential confounding effect, the following covariates were included in the regression models: age group, education, marital status, familial income, working at the time of the study, religion (as declared), migration (not born in the cities where the study was conducted), city: São Paulo or Rio de Janeiro, self-perception of intensity of trauma (evaluated with a five-point Likert scale and stratified as 1–3: low/moderate; 4–5: severe).

### Statistical analysis

Initially, the frequency distributions were examined for the independent variables and covariates, stratified by city. Logistic regression models were then fitted for resilience and independent variables to estimate the incidence density ratio. As shown by Pearce [37] and Reichenheim and Coutinho [38], the exponential of the logistic regression coefficient can estimate the incidence density ratio when the following conditions are met: (i) The population must be in a steady state over the study period (stationary). (ii) No selective survival is allowable. (iii) The exposure may not influence the survival or recovery probabilities. (iv) No reverse causality is allowed. (v) The temporal directionality from the exposure to the outcome must be sustainable, either theoretically or by means of a thorough data collection procedure.

To determine statistical significance in the bivariate analyses, we used the chi-square test for categorical variables, the Student *t*-test for continuous variables that were normally distributed, and the Kruskal-Wallis (KW) test for continuous variables that were skewed. Variables with *p*-values less than 0.20 were initially selected for inclusion in multivariate models. Multiple logistic regression models were fitted to investigate the contribution of the variables to resilience and to control for potential confounders. We entered these variables in the multivariate logistic model using a stepwise strategy. This procedure was not automatic, but was controlled by the authors. The order of entry of the variables was defined based on their *p*-values. Variables with greater statistical significance (lower *p*-values) were entered first in the model. Variables with *p*-values less than 0.10 were retained in the model. Covariates were considered confounding factors if their inclusion in the model produced a 20% change in the magnitude of the exposure estimate. Interaction between parental mental health and variables related to traumatic events were tested by likelihood ratio test.

To explore the role of positive and negative affect on resilience, we conducted two additional analyses including either PA or NA variables as dependent variables in linear regression models. The first one included two independent variables related to trauma: intensity of the worst trauma, and a new variable that combined information on trauma intensity and resilience in five categories: no trauma, moderate trauma without resilience, moderate trauma with resilience, severe trauma without resilience, severe trauma with resilience. The second analysis included the number of different types of trauma, resilience and an interaction term (*number of different types of trauma X resilience*). Statistical analysis was performed using Stata 10.0.

### Ethical issues

Participants were informed about research procedures and risks and signed an informed consent form that was submitted and approved by the Ethical Committee of the Federal University of São Paulo. A telephone number was provided for participants who wanted to be seen by a mental health professional.

## Results

Overall, 2,159 of the participants were from the city of São Paulo and 1,072 from the city of Rio de Janeiro. There was a slight predominance of women within the studied populations. Caucasian was the main ethnic group, followed by mixed-race groups. One-third of the interviewed subjects mentioned some kind of traumatic event during childhood. Table 1 displays the characteristics of the individuals exposed to traumatic events, stratified by city of residence.

**Table 1:** **Characteristics of the sample, by city: São Paulo (n = 2159); Rio de Janeiro (n = 1072)**

**Variables**	**São Paulo**	**Rio de Janeiro**
	**n (%)**	**n (%)**
**Gender**		
Female	1205 (55.8)	597 (55.7)
Male	954 (44.2)	475 (44.3)
**Age group (years)**		
15-24	399 (18.5)	171 (16.0)
25-34	619 (28.8)	220 (20.5)
35-44	439 (20.3)	221 (20.6)
45-54	333 (15.4)	206 (19.2)
55-64	234 (10.8)	140 (13.1)
65-75	135 (6.2)	114 (10.6)
**Ethnic group**		
White	970 (45.0)	457 (42.7)
Black	295 (13.7)	205 (19.2)
Mixed-race	811 (37.6)	365 (34.1)
Asian	50 (2.3)	23 (2.1)
Indigenous	19 (0.9)	15 (1.4)
Other	12 (0.5)	5 (0.5)
**Education (years)**		
Illiterate	70 (3.2)	14 (1.3)
1-4	397 (18.4)	124 (11.6)
5-8	597 (27.7)	246 (22.9)
9-12	832 (38.5)	453 (42.3)
13 or more	263 (12.2)	235 (21.9)
**Marital status**		
Single	585 (27.1)	314 (29.3)
Married	1253 (58.0)	577 (53.8)
Widowed	108 (5.0)	64(6.0)
Divorced	213 (9.9)	117 (10.9)
**Family income (minimum wage)** ^**a**^		
Less than 2	780 (40.3)	280 (29.8)
2 - 3.9	593 (30.6)	319 (33.9)
4 or more	565 (29.1)	342 (36.3)
**Working (yes)**	1341 (62.1)	641 (59.8)
**Religion**		
Catholic	1299 (60.6)	573 (53.8)
Spiritism	94 (4.4)	94 (8.8)
Evangelical	531 (24.7)	270 (25.4)
Other	41 (1.9)	24 (2.3)
Spirituality without religion	151 (7.0)	80 (7.5)
Atheist	30 (1.4)	23 (2.2)
**Migration (yes)**	1142 (52.9)	330 (30.8)
**Parental mental disorders**	137 (6.4)	87 (8.1)
**Childhood traumatic event**		
None	1374 (63.6)	652 (60.8)
Trauma not physical or sexual	608 (26.2)	315 (29.4)
Physical abuse	132 (6.1)	76 (7.1)
Sexual abuse	45 (2.1)	29 (2.7)

^a^ US$268 at the time of study. Missing information in São Paulo and Rio de Janeiro are 23 and 22%, respectively.

Bivariate analyses of the relationship between demographic and psychological variables, and resilient outcomes after a traumatic event are summarized in Table 2. Among the independent variables, resilience was found to be positively associated with male gender and absence of parental mental disorder, and inversely associated with indigenous ethnic groups and childhood traumatic events. With regard to other covariates, there was an association of resilience with age, education, marital status, and family income.

**Table 2:** **Bivariate analysis of demographics, psychological variables and resilience**

**Independent variables**	**Resilient**	***χ*** ^**2**^ **(** ***df)***	**p-value**
	**n (%)**		
**Gender**			
Female	641 (35.6)	74.4 (1)	< 0.001
Male	727 (50.7)		
**Ethnic group** ^**a**^			
White	608 (42.6)		
Black	208 (41.6)		
Mixed-Race	500 (42.5)	5.9 (5)	0.32
Asian	31 (42.5)		
Indigenous	8 (23.5)		
Other	9 (52.9)		
**Parental mental disorders**			
(mother and/or father)			
No	1300 (43.2)	17.3 (1)	< 0.001
Yes	65 (29.0)		
**Childhood traumatic event**			
None	971 (47.9)		
Trauma not physical or sexual	335 (36.3)	99.5 (3)	< 0.001
Physical abuse	53 (25.5)		
Sexual abuse	6 (8.1)		
**Age group (years)**			
15-24	258 (45.3)		
25-34	315 (37.5)		
35-44	268 (40.6)	26.3(5)	< 0.001
45-54	226 (41.9)		
55-64	162 (43.3)		
65-75	136 (54.6)		
**Education (years)**			
Illiterate	38 (45.2)		
1-4	208 (39.9)		
5-8	325 (38.5)	11.2(4)	0.03
9-12	562 (43.7)		
13 or more	232 (46.6)		
**Marital status**			
Single	404 (44.9)		
Married	780 (42.6)	14.3 (3)	0.003
Widowed	72 (41.9)		
Divorced	109 (33.0)		
**Working**			
No	535 (42.8)	0.29(1)	0.59
Yes	830 (41.9)		
**Migration**			
No	762 (43.3)	1.8 (1)	0.18
Yes	603 (41.0)		
**Religion**			
Catholic	834 (44.5)		
Spiritism	75 (39.9)		
Evangelical	318 (39.7)	10.7 (5)	0.06
Other	24 (36.9)		
Spirituality without religion	84 (36.4)		
Atheist	22 (41.5)		
**Family income (minimum wage)**			
Less than 2	397 (37.5)		
2 - 3.9	409 (44.9)	11.5 (2)	0.003
4 or more	383 (42.2)		

^**a**^Indigenous versus other: *χ*2 = 4.9, df = 1, p = 0.03.

When the four independent variables, presenting p-values less than 0.20 in bivariate analysis, were entered one by one in a multivariate logistic regression model, only ethnicity lost its association with resilience (Table 3). The incidence rate among men was 34% higher than among women, and men were still more resilient even after controlling for type of trauma (31% higher). We found an inverse “dose–response” or linear relationship between childhood violence and resilience. Those individuals who had childhood trauma were less likely to adapt positively if the trauma was physical abuse, and even less if the major trauma was sexual abuse. In this last case, resilience was reduced to 81%, compared with participants without trauma during childhood, but the precision of this estimate is not very high. Those participants who reported no parental mental disorders showed a 35% higher incidence of resilience compared with individuals who had at least one parent who suffered from a mental disorder. No interactions were found between parental mental health and any variable related to the traumatic event.

**Table 3:** **Logistic regression of resilience by pre-mental disorder variables**

**Independent variables**	**IDR** ^**a**^	**CI (95%)**	**p-value**
**Gender (male)**	1.34	1.14 – 1.58	< 0.001
**Ethnic group**			
White	Reference		
Black	0.98	0.77 – 1.25	0.88
Mixed-race	1.06	0.89 – 1.28	0.50
Asian	1.12	0.66 – 1.92	0.67
Indigenous	0.47	0.19 – 1.15	0.10
Other	2.41	0.74 – 7.89	0.15
**Childhood traumatic event**			
None	Reference		
Trauma not physical or sexual	0.67	0.56 – 0.81	< 0.001
Physical abuse	0.53	0.37 – 0.76	0.001
Sexual abuse	0.19	0.08 – 0.46	< 0.001
**No parental mental disorders**	1.35	0.96 – 1.89	0.07
**Positive activation (“Positive Affect”)**	0.99	0.96 – 1.03	0.63
**Negative activation (“Negative Affect”)**	0.83	0.78 – 0.88	< 0.001
**Interaction term (“Positive Affect” X “Negative Affect”)**	1.002	1.000 – 1.003	0.10

*Note:* Likelihood ratio *χ* ^2^ = 733.9, *df = 30.*

^a^Adjusted by age group, marital status, educational level and religion.

To explore the way resilience may operate, we investigated the association of resilience with positive and negative affect, as measured with the PANAS. In bivariate analyses, resilient individuals had lower average scores on negative affect (NA) than non-resilient individuals (17.3 versus 23.6, KW = 554.8, *df* = 1, *p* < 0.01). No difference was found for the average positive affect (PA) scores (32.7 versus 32.8, KW = 0.05, *df* = 1, *p* = 0.89). Nevertheless, when those variables were included in the multivariate model, a small interaction between PA and NA was found in the sample, with a marginal significance level. This finding suggests that at the lowest level of PA, each point increment in the NA score reduces the chance of being resilient in 17%. However, as PA scores increase, the negative effect of NA over resilience decreases, suggesting the possibility of a buffering effect of PA over NA. At 40 points of PA, for example, the reduction in the chance of being resilient becomes 12% for each increment in NA score.

**Figure 1:**
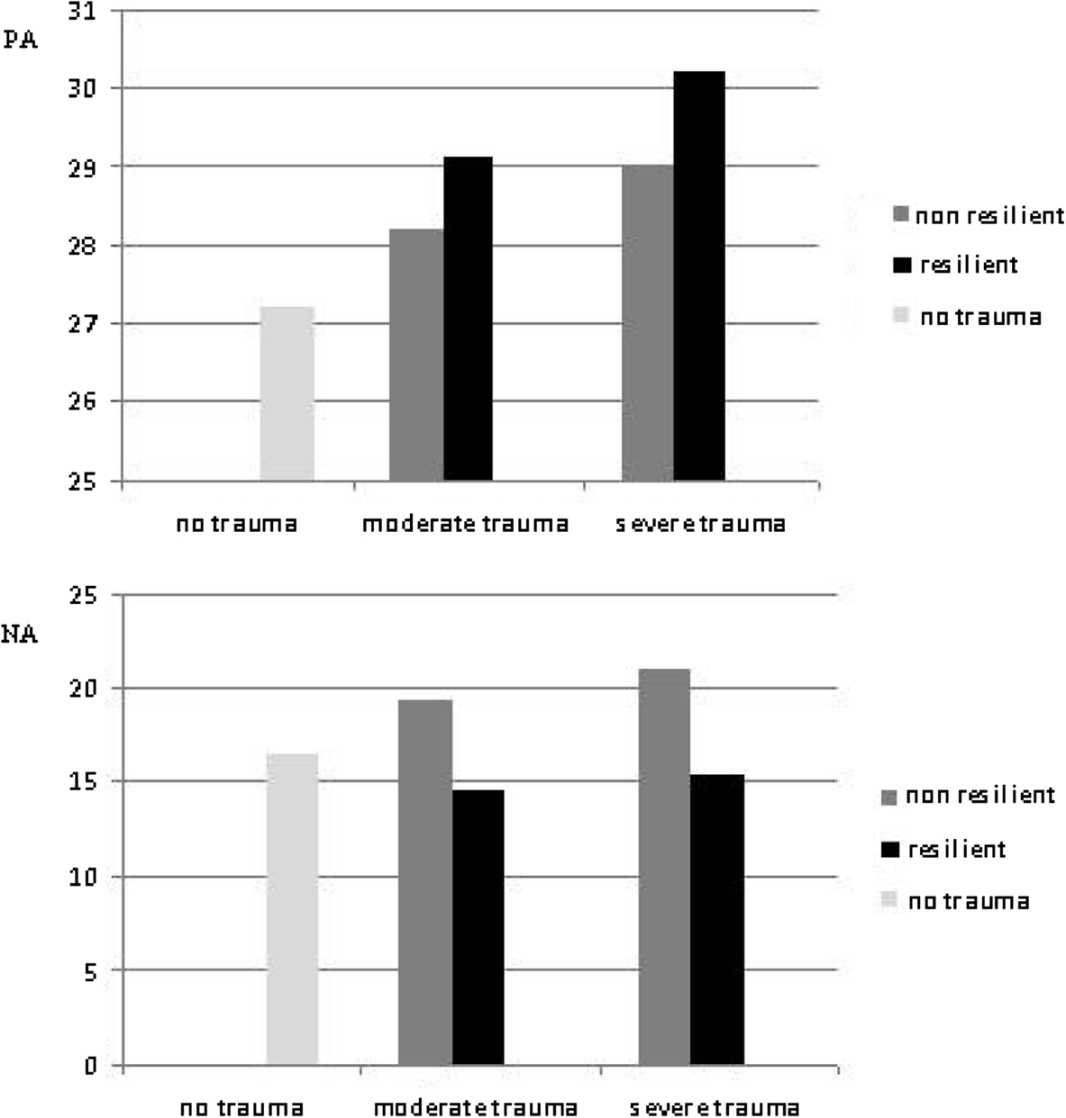
**Positive affect (PA) and negative affect (NA), trauma intensity and resilience.** Average scores of PA and NA according to combined categories of trauma intensity and resilience (adjusted by gender, age, affect, parental mental disorder and childhood trauma).

**Figure 2:**
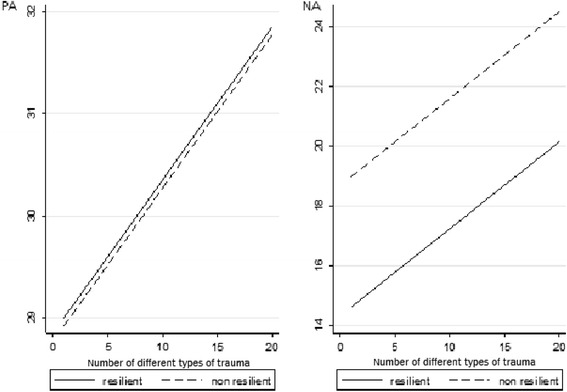
**Positive affect (PA) and negative affect (NA), number of different types of trauma and resilience.** Average scores of PA and NA according to combined categories of the number of different types of trauma and resilience (adjusted by gender, age, affect, parental mental disorder and childhood trauma).

As some protective effects may only play their role if activated by risk or in response to adversity, we investigated the possible influence of traumatic experience on positive affect, besides the expected emergence of negative affect. Figure 1 presents the average scores of positive affect for the combination of trauma intensity and resilience, controlling for negative affect, gender, age, parental mental disorder and childhood trauma. Figure 1 shows the same analysis for negative affect. It seems that the presence of trauma influences positive affect by increasing its score in a linear pattern. It is also important to note that within each category of trauma intensity, resilience is associated with higher positive affect. Conversely, negative affect shows a different pattern. Although its average score also increases with trauma intensity, resilient individuals are associated with lower negative affect. We found the same pattern with the variable diversity of trauma, i.e., exposure to different types of trauma, as shown in Figure 2. These figures were based on data presented in Tables 4, 5, 6 and 7.

**Table 4:** **Average scores of Positive Affect (PA) according to combined categories of trauma intensity and resilience**

**PA**	**Beta coefficient**	**Standard error**	**p-value**
Constant	27.24	0.56	< 0.01
Moderate trauma non-resilient	1.02	0.67	0.13
Moderate trauma resilient	1.83	0.52	< 0.01
Intense trauma non-resilient	1.75	0.38	< 0.01
Intense trauma resilient	2.98	0.39	< 0.01
**Covariables**			
Gender	0.75	0.24	0.002
Age	– 0.09	0.008	0.22
Negative affect	0.17	0.017	< 0.01
Parental mental disorder	– 0.73	0.47	0.12
childhood trauma	– 0.01	0.18	0.97

R^2^ = 0.05.

**Table 5:** **Average scores of Negative Affect (NA) according to combined categories of trauma intensity and resilience**

**NA**	**Beta coefficient**	**Standard error**	**p-value**
Constant	16.21	0.65	< 0.01
Moderate trauma non-resilient	2.77	0.65	< 0.01
Moderate trauma resilient	−2.00	0.50	< 0.01
Intense trauma non-resilient	4.41	0.36	< 0.01
Intense trauma resilient	−1.15	0.38	<0.01
**Covariables**			
Gender	−2.15	0.23	<0.01
Age	−0.05	0.01	<0.01
Positive affect	0.16	0.16	<0.01
Parental mental disorder	1.06	0.45	0.02
Childhood trauma	0.90	0.17	<0.01

R^2^ = 0.22.

**Table 6:** **Average scores of Positive Affect (PA) according to combined categories of number of different types of trauma and resilience**

**PA**	**Coefficient**	**Standard error**	**p-value**
Constant	28.77	0.62	< 0.01
Number of different types of trauma	0.15	0.06	< 0.01
Resilience	−0.36	0.46	0.44
Interaction term*	0.44	0.10	< 0.01
**Covariables**			
Gender	0.54	0.25	0.04
Age	−0.01	0.01	0.06
Negative affect	0.17	0.02	< 0.01
Parental mental disorder	−0.87	0.48	0.07
Childhood trauma	−0.34	0.19	0.07

*Number of different types of trauma x resilient.

R^2^ = 0.05.

**Table 7:** **Average scores of Negative Affect (NA) according to combined categories of number of different types of trauma and resilience**

**NA**	**Coefficient**	**Standard error**	**p-value**
Constant	18.70	0.72	< 0.01
Number of different types of trauma	0.29	0.06	< 0.01
Resilience	−4.38	0.45	< 0.01
Interaction term*	−0.26	0.10	< 0.01
**Covariables**			
Gender	−2.13	0.25	<0.01
Age	−0.04	0.01	<0.01
Positive affect	0.16	0.02	<0.01
Parental mental disorder	0.80	0.48	0.09
Childhood trauma	0.68	0.19	<0.01

*Number of different types of trauma x resilient.

R^2^ = 0.23.

**Figure 3:**
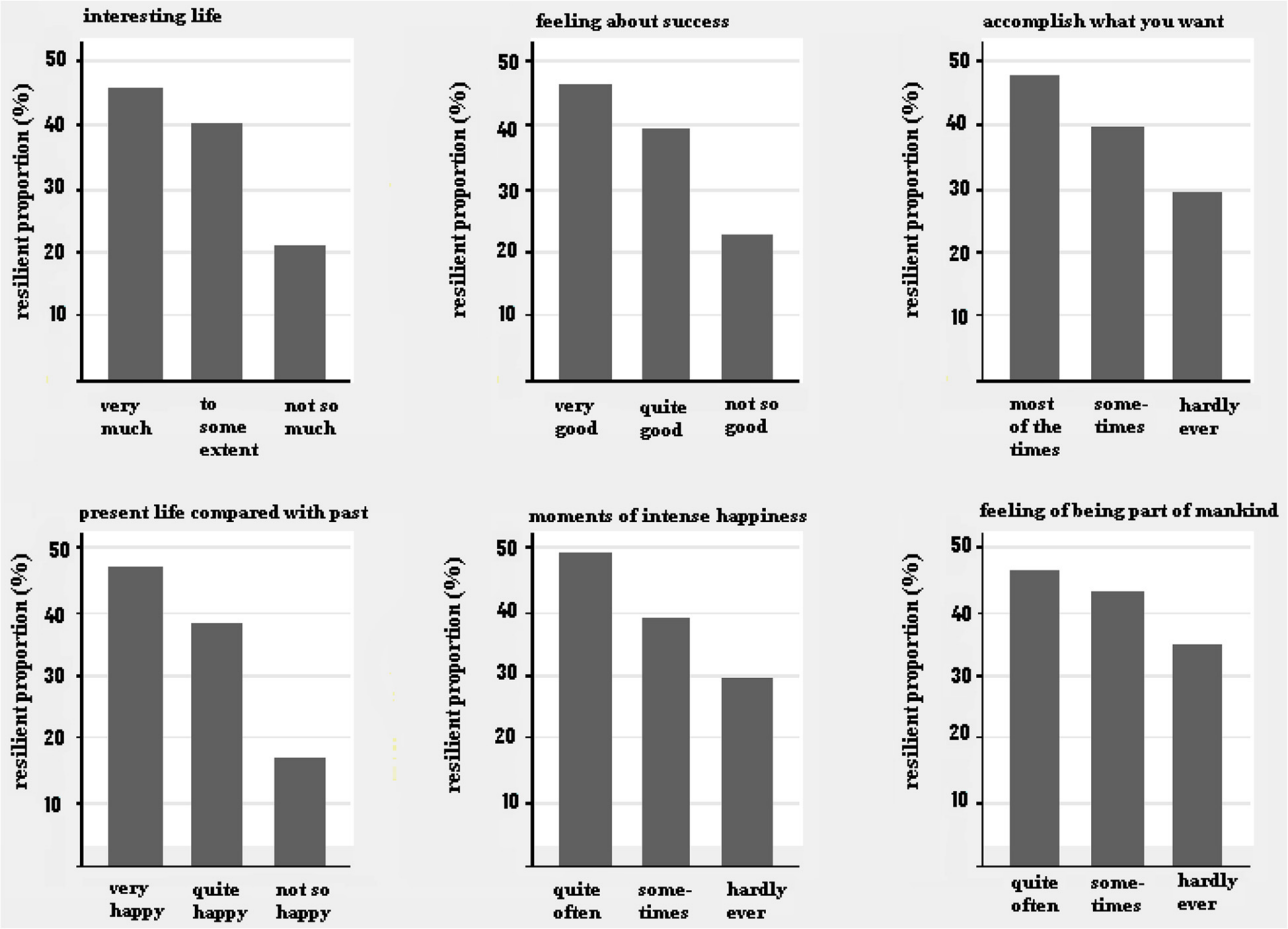
**Proportion of resilience (%) by items related to well-being.**

Finally, we found a higher proportion of “resilient” individuals among those participants with the highest scores on each item related to well-being (Figure 3).

## Discussion

Our findings support the hypotheses that resilience may be influenced by a set of individual and social factors. We found that among those participants who experienced a traumatic event, there was a higher proportion that adapted positively among men. It is well known that common mental disorders, such as major depression and PTSD, are more likely to occur in women than men in adult life [39,40]. Some authors have suggested that gender differences in PTSD prevalence could be a function of the type of traumatic events to which women are exposed (such as sexual assault) [39]. In our sample, men were still more resilient even when controlling for sexual trauma. Also, Kendler et al. [41] could not attribute the lower prevalence of major depression in men versus women, to a difference in the rates of reported stressful life events, or to differential sensitivity to the pathogenic effect of such events.

It is also notable that the present study did not include conduct disorders within the profile of investigated diagnoses, which are more common in men [40]. Nevertheless, as alcohol related disorders are a frequent pathological reaction to traumatic events in the male population [42], and are commonly associated with other externalized problems [43,44], we believe that its inclusion in this study may have overcome at least part of this limitation. Moreover, individuals in our sample were older than 15 years of age and the literature on developmental psychopathology indicates that at higher levels of development (in terms of maturity), pathology tends to be expressed more often in internalizing symptoms, rather than in externalizing behavioral disturbance [22].

In our sample, the indigenous group showed the smallest proportion of resilience, but this finding did not reach statistical significance. As the number of individuals in this ethnic group was small (n = 34), we cannot rule out that this finding was caused by the lack of statistical power. Other studies have found a risk of adverse mental health outcomes in minority ethnic groups, but some have suggested that this risk is more likely to occur in areas where this minority is smaller [45].

Consistent with the literature, individuals in our sample with a history of childhood trauma showed a smaller chance of having positive adaptation [20,46,47], especially if that trauma was abuse. Individuals with a history of sexual abuse during childhood showed a very small chance of resilience. The inverse linear relationship found between childhood trauma and resilience reinforces the idea of causality. As the majority of children who suffer maltreatment are adversely affected, child abuse is considered the greatest failure of the environment to provide opportunities for normal development [48]. Our data reinforce the importance of developing preventive and health promotion interventions in childhood, justifying the allocation of resources for such interventions.

Moreover, individuals without a history of parental mental disorder were more likely to be resilient when exposed to a traumatic event. The occurrence of positive adaptation to a traumatic event increased by 48%, in those participants without a history of parental mental disorders. After adjusting for childhood violence, the influence of absence of parental disorders was weakened (35%) and its significance became marginal (*p* = 0.07). These findings may be a result of the interplay between biological factors, such as heredity, and environmental influences, such as parental bonding and parenting style [49]. These factors may play a mediating role between parental psychopathology and child symptoms of disorder. For example, children with psychiatrically ill parents who were not exposed to parental maltreatment may show very low levels of both internalizing and externalizing problems, compared with similar children exposed to such maltreatment [50]. Also, Luthar and Brown [20] showed that differences in parenting behaviors mediate the influence of maternal depression and/or maternal drug abuse on offspring psychopathology.

Positive parenting behavior may also moderate the effect of temperamental dispositions on later adjustment [50]. Silk et al. [51] demonstrated high levels of parent–child relationship quality were associated with high positive adjustment for children, except for those experiencing high or chronic neighborhood risks. An adversity, such as violence or an economic crisis in parents’ lives, could undermine the adaptation of a child through their indirect effects on functioning, mood or parental distress [12,52], although a meta-analysis demonstrated that parenting accounted for only 4% of the variance in child anxiety [53]. Our study did not find interactions between parental mental health and any variable related to the traumatic event. It is important to remember that we examined perceived parental mental illness, and there may be a substantial gap between representations of parents and the way they actually behave [54].

It is also well known that early secure attachment enhances self-esteem and self-efficacy and that the roots of optimism lie in infancy, when the child may develop the confidence that he or she and the environment will be able to manage any problem [55]. It is also believed that immediate and long-term cognitive, social, behavioral, and even health benefits associated with attachment security might be partially mediated by the capacity to experience positive emotions [56].

It seems that successful emotional regulation involves the dynamic and coordinated interplay between positive and negative emotional states, and individuals who display positive emotions and derive positive meaning from adversities report more resilience [23,25,56].

From our data, we noticed that both PA and NA increased with trauma intensity in a linear manner. We also found that although both PA and NA increased with trauma intensity, their patterns were different. For each trauma intensity category, PA was higher among those participants with positive adaptation compared with those without it. Conversely, NA followed an inverse pattern. Moreover, PA seems to function as a resilience factor, as a moderator factor that is activated by trauma and buffers NA.

These findings are consistent with Diamond and Aspinwall’s [56] suggestion that some of the most important effects of positive emotions may occur in interaction with negative emotional states, as they prevent acute episodes of negative affect from becoming solidified into defensive and maladaptive regulatory patterns. With respect to emotional regulation, they consider that the optimal development outcome is a dynamic flexibility in emotional experience, and a co-activation of negative and positive emotions seems to be implicated in a resilience process.

Positive emotions promote the reduction of physiological arousal produced by negative emotions [26,27] and help to buffer against stress [57]. According to Tugade et al. [26], positive emotions are crucial for enhancing coping mechanisms in the face of adversity, contributing to psychological and physical well-being and protecting against depressive mood. Experimental studies have found that positive affect increases cognitive flexibility in ways that promote effective decision-making and problem-solving strategies, with the development of lasting knowledge and other personal abilities and social resources [56,57].

In a study of 279 female twin pairs, Wichers et al. [58] demonstrated that positive emotions not only buffer against NA reactivity but additionally attenuate genetic effects on bias of negative mood in daily life. The authors suggest that genes that render individuals vulnerable to depression are expressed in part through increases in NA reactivity to stress. “However, this process is not deterministic and can apparently be moderated when subjects are able to co-experience higher levels of positive emotions alongside the increases of NA after stressful events,” according to Wichers et al., p.455 [58]. Little research exists examining the role of PA as a protective factor in NA reactivity.

Some studies have found a negative correlation between the NA and PA scales [31,34]. But, surprisingly, these dimensions were positively correlated (*r* = 0.15; *p* < 0.001) in our sample, which was exposed to traumatic events. As shown in Figures 1 and 2, it seems that trauma may activate both dimensions concomitantly. Our results also suggest that resilience may depend by the amount of positive affect that buffers against negative affect.

We constructed our dependent variable *resilient* as the absence of PTSD, depression, anxiety, and alcohol disorders, without including an examination of mania or hypomania disorders. Thus, the possibility of at least some misclassification of individuals with manic or hypomanic symptoms as resilient may have occurred. Nevertheless, considering the common comorbidity with depression, anxiety or alcohol disorders, and the lower prevalence of bipolar disorders, we believe that the pattern of correlation of positive affects and resilience shown in our study may represent a general characteristic of our sample.

Misclassification of some individuals as resilient may also have occurred as we did not investigate other diagnoses, such as disorders related to other substances, psychoses or somatoform disorders. Psychoses are not common in the general population and are much less frequently associated with traumatic events than the disorders studied here. The investigation of somatoform disorders was not possible owing to the difficulty of establishing a differential diagnosis with other disorders of organic etiology. However, these individuals are likely to have been classified in the category “not resilient”, given the presence of the comorbidities investigated.

Despite these limitations, a higher proportion of “resilient” individuals among participants with the highest scores on items related to well-being was found, suggesting that the criteria for classifying positive adaptation used in this study achieved a degree of convergent validity.

Establishing a relationship between the time of occurrence of the traumatic event and the time of onset of the psychiatric disorders was not able to be achieved. Thus, some people classified as having a “negative adaptation”, may have had a previous diagnosis, without triggering any other response after the trauma. Whereas resilience has a dynamic character, in these cases, the positive adaptation to a given trauma may be underestimated because the individual had a diagnosis at some other time in their life. This methodology has been used in another study [59] and has an exploratory character.

Causal inference in cross-sectional design can be affected by reverse causality. Although our focus was on investigating how the influence of variables more likely to appear early in the history of individuals, the hypothesis of parental mental illness secondary to a child’s disorder cannot be ruled out. Nevertheless, the exclusion of individuals with mental retardation and others who could not respond appropriately to the interview may have minimized this possibility. It is important to mention the possibility of a bidirectional interactive process between these variables [50] that could not be investigated by a cross-sectional study design. The possibility that scores on positive and negative affect, rather than being predictors of psychopathology, may be influenced by a current mental problem cannot be excluded. However, results in a longitudinal cohort conducted by Watson et al. [60] indicated that trait measures of PANAS show temporal stability, even across retest intervals as long as 7.5 years, with a little more variable result for negative affect than for positive affect. Their study has also shown that this trait affect scale has predictive validity, with scores on the PANAS being significantly related to measures of current anxious and depressive symptomatology that were completed several years later.

## Conclusion

This was the first epidemiological study in Brazil that investigated resilience to traumatic events and attempted to identify how affect operates to achieve positive adaptation. The impact of parental mental diseases and childhood violence on resilience suggested by this current study can improve efforts in finding preventive and health-promoting intervention strategies for parenting, and support the allocation of resources for those interventions. Moreover, the possibility of a moderating role of positive affect that is becoming increasingly evident in the literature, also expands the potential for preventive and health promotion interventions for individuals exposed to traumatic events.
